# Cystic cavernous malformation of the cerebellopontine angle: Case report and literature review

**DOI:** 10.1186/1477-7819-9-36

**Published:** 2011-03-23

**Authors:** Haiyan Huang, Kan Xu, Limei Qu, Ye Li, Jinlu Yu

**Affiliations:** 1 Department of Neurosurgery, The First Hospital of Jilin University, 71 Xinmin Avenue, Changchun 130021, PR China; 2 Department of Pathology, The First Hospital of Jilin University, 71 Xinmin Avenue, Changchun 130021, PR China; 3 Department of Radiology, The First Hospital of Jilin University, 71 Xinmin Avenue, Changchun 130021, PR China

## Abstract

**Background:**

Cavernous malformations (CMs) in the cerebellopontine angle (CPA) are rare, and most of such CMs reported to date are solid and extend from the internal auditory canal into the CPA. In contrast, cystic CMs that arise in the CPA and do not involve the internal auditory canal and dura of the skull base are extremely rare.

**Case presentation:**

A 50-year-old man presented with vertigo and progressive hearing loss in the right ear. MRI examination revealed a lesion in the CPA with solid and cystic components. Surgery was performed. Well-circumscribed adhesion to cranial nerves, the cerebellum, or the brain stem was noted during surgery. The lesion was totally resected. Pathological examination suggested the lesion to be a CM. At 1-year follow-up, the symptoms at presentation had resolved and no complications had occurred.

**Conclusion:**

Although cystic CMs of the CPA have no established imaging features, a diagnosis of CMs may be suspected when a cystic lesion is present in the CPA and does not involve internal acoustic meatus or dura mater of the skull base. Skillful microsurgical techniques and monitoring of cranial nerves will secure good outcomes for patients with cystic CMs in the CPA.

## Background

Cavernous malformations (CMs) occur in 0.4-0.8% of the general population, and they account for 10-15% of all vascular malformations of the central nervous system [1,2]. Intracranial CMs are commonly located in the supratentorial region, brain stem, basal ganglion, and cerebellar hemisphere [3]. However, CMs arising in the cerebellopontine angle (CPA) are an extremely rare clinical entity. At present, there are few reports available on such CMs. The majority of the CMs in the CPA reported to date are solid lesions that arise from the internal auditory canal and extend to the CPA [4]. In contrast, cystic CMs in the CPA are very uncommon: Only four cases of cystic CMs in the CPA have been reported to date, and none involved the internal auditory canal [5-8]. The exact causes of cyst formation remain largely undefined; however, previous studies have suggested that recurrent minor hemorrhage from the sinusoids of the vascular malformation or from the neocapillary of the cyst wall may underlie the growth of the cyst [8,9]. Herein, we describe a patient with cystic CM of the CPA who was admitted to our hospital and whose lesion was not adherent to the internal auditory canal or dura of the skull base, together with four similar cases identified through a literature search. Our goal was to summarize the clinical, radiological, and treatment features of CMs of the CPA.

### Case Presentation

**Figure 1:**
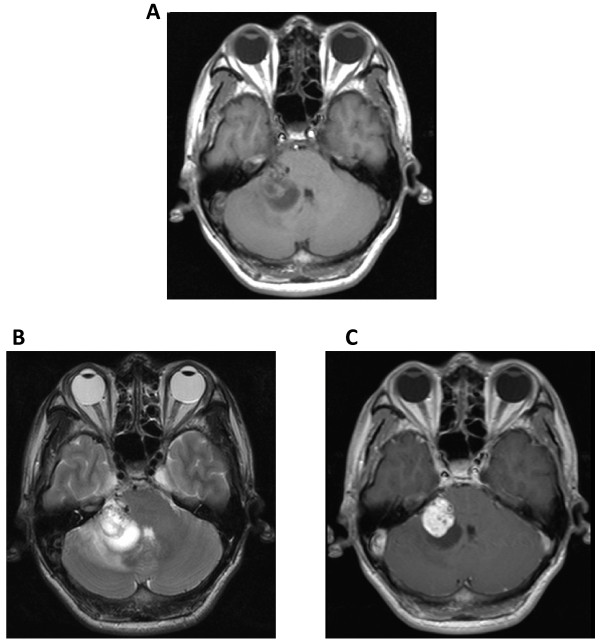
**MRI showed a solid cystic lesion in the right CPA that compressed both the brain stem and the cerebellum**. The anterior portion of the lesion was solid and showed signs of cystic changes, and the posterior portion of the lesion was cystic. The size of the solid component was about 2.2 cm × 2.2 cm × 2.3 cm. The solid component showed hyper- and isointensity on T1WI images (A) and mixed intensity on T2WI images (B), and it was significantly enhanced after contrast administration (C).

**Figure 2:**
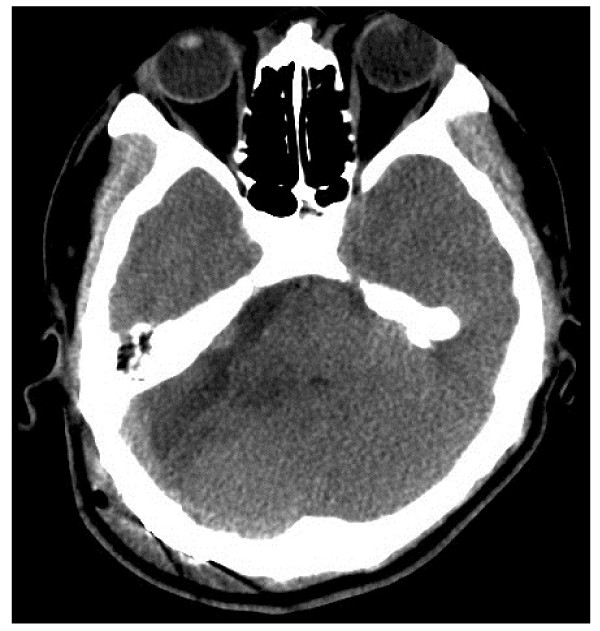
**Postoperative CT examination showed that the CMs had been completely removed**.

**Figure 3:**
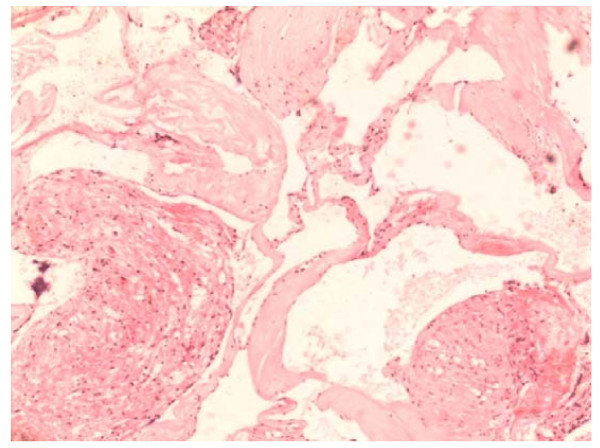
**Photomicrograph showing the dense clusters of thin-walled cavernous vascular channels separated by collagenous septae without any intervening neural tissues**. (Original magnification 200 ×).

A 50-year-old man presented with progressive hearing loss in the right ear and vertigo for the past 6 months and facial numbness and unsteady gait for the past 15 days. Upon physical examination, he was found to have right ear sensory hearing loss, ataxia, diminished sensation in the right face (supplied by the third branch of the trigeminal nerve), and high frequency hearing loss in the right ear, as revealed by brain stem auditory evoked potential examination. MRI examination revealed a lesion in the CPA with solid and cystic components, which compressed the brain stem and the cerebellum. The anterior portion of the lesion was solid and showed signs of cystic changes, whereas the posterior portion of the lesion was cystic. The solid component of the lesion showed hyper- and isointensity on T1WI images and mixed hyper- and hypointensity on T2WI images, and it was significantly enhanced after contrast administration. The size of the solid component of the lesion was about 2.2 cm × 2.2 cm × 2.3 cm (Figure 1). Surgery was performed via a right suboccipital retrosigmoid approach, and intraoperative monitoring of cranial nerves was conducted. The lesion was revealed to be red, well margined, firm, vascular, anteriorly solid with cystic changes, and adherent to the brain stem and the cerebellar hemisphere, the trigeminal nerve, and facial and acoustic nerves. Following separation of the lesion from adjacent nerves and tissues along the border of the lesion under microscopy, the lesion was totally resected in a partitioning manner. The xanthochromic fluid in the back of the lesion was drained during surgery. The patient recovered well after surgery and presenting symptoms were significantly relieved. Postoperative CT scans demonstrated that the lesion was completely resected (Figure 2). The histopathological features of the lesion were consistent with a CM (Figure 3). At 1-year follow-up, this patient's symptoms at presentation had resolved.

## Discussion

Locations of CMs are primarily associated with the volume of the brain tissues; therefore, CMs are more common in supratentorial areas and occasionally are found in the brain stem, the cerebellum, cranial nerves, dura mater, and venous sinuses [3,10]. However, the occurrence of CMs in the CPA is rare, and most of such CMs reported to date are solid and extend from the internal auditory canal into the CPA [4]. In contrast, CMs that arise in the CPA and do not involve the internal auditory canal and dura of the skull base are extremely rare. To date, only four such cases have been reported [5-8]. (Table 1). In fact, CMs arising in the central nervous system are mostly solid, and cystic CMs are rare. In the present study, we describe an additional case of cystic CMs in the CPA (treated in our hospital), together with the four similar cases previously reported.

**Table 1:** Clinical data for the five cases of cystic CMs in the CPA

NO	Author/Year	Age/Sex	History	Symptoms	Radiological findings	Surgical findings	Outcome
1	Iplikçioğlu/1986 [5]	30/Male	7 years	Hearing loss, facial palsy, facial sensory loss, headache	CT: solid cystic lesion with a large cyst and small nodules; slight enhancement of cyst wall; calcification within the nodules	Bluish-gray lesion with xanthochromic fluid. The lesion was adherent to the brain stem and 7^th ^and 8^th ^cranial nerves. The lesion did not have a rich blood supply.	Symptoms were not resolved and left facial palsy and hearing loss persisted.

2	Brunori/1996 [6]	60/Male	2 months	Facial sensory loss, tinnitus, vertigo, ataxia	MRI: solid cystic lesion with multiple cysts; marked enhancement of the solid component; hemosiderin deposition rim bordering the lesion	Reddish-blue, mulberry like lesion with xanthochromic fluid. The lesion was adherent to the brain stem and 7^th ^and 8^th ^cranial nerves. The lesion had a rich blood supply.	The patient died due to massive hemorrhage on the third postoperative day.

3	Vajramani/1998 [7]	46/Male	7 months	Headache, tinnitus, vertigo, hearing loss, right cerebellar signs	CT and MRI: solid cystic lesion with a large cyst and small nodules; the nodules were enhanced after contrast administration	Red lesion with xanthochromic fluid. The lesion was adherent to the brain stem and was excised in two stages. The lesion had a rich blood supply.	Symptoms were not resolved but no complications developed.

4	Stevenson/2005 [8]	57/Male	Not available	Hearing loss, tinnitus, facial numbness and facial sensory loss, ataxia	MRI: solid cystic lesion a large cyst; the cystic wall was enhanced	Lobulated lesion with xanthochromic fluid. The lesion was adherent to the brain stem and the 5^th^, 7^th^-11^th ^cranial nerves. The lesion had a rich blood supply.	Good recovery.

5	Present case/2010	50/Male	6 months	Impaired hearing, vertigo, ataxia, facial numbness	MRI: solid cystic lesion with a posterior cystic component; marked enhancement of the solid component on contrast-enhanced MRI	Red lesion adherent to the brainstem, cerebellum, and 5^th^, 7^th^, and 8^th ^cranial nerves. The lesion had a rich blood supply.	Good prognosis

A retrospective analysis of the imaging features of the five cases revealed that cystic CMs in the CPA had unspecific imaging manifestations. Of the five cases, four had large cysts and small nodules and one case had multiple cysts interspersed in the solid component of the lesion. Enhancement of varying degrees was noted in all five cases. These findings are consistent with the imaging features of 25 cases of cystic CMs reviewed by Ohba [9]. Our study also confirms that cystic CMs arising in the CPA are rare cystic CMs in the central nervous system. Only four such cases (16%) were found among the 25 cases of cystic CMs reviewed by Ohba [9]. Herein we reviewed a relatively large series of cystic CMs in the CPA, including one case encountered in our institution, in an attempt to outline the clinical and therapeutic characteristics of cystic CMs in the CPA.

The causes of cystic degeneration of CMs remain unknown. Recurrent minor hemorrhage of internal vascular sinuses or neocapillaries within CMs may be involved in the process. When bleeding episodes occur within a CM, the osmotic pressure across the CM membrane changes, leading to gradual fluid accumulation within the CM and cystic degeneration, followed by CM growth [9,11,12]. Cystic degeneration within the CMs in the CPA is a progressive process, thus CMs may be at different stages of cystic degeneration when imaging examinations are performed. Consequently, the CMs may show various features of cystic degeneration. For example, multiple cysts may be seen within the solid component of the CM, and a large cyst may be seen in combination with small nodules. In addition, cystic CMs may have different blood supply profiles. All of these features contribute to different enhancement patterns upon contrast-enhanced CT or MRI examination, which can vary from no enhancement at all to marked enhancement. Solid CMs in the brain can show specific MRI manifestations (e.g., a hypointense rim containing hemosiderin deposits on T2WI or DWI sequences) [13,14].

However, out of the five cases described in this report, only one case showed a rim of hemosiderin deposition. Cystic degeneration is less severe in small CMs, which are mostly solid. The characteristic hemosiderin deposition rim may be caused by the exudated blood from a hemorrhage, which cannot enter the inside of the CM. Because of the complex imaging features of cystic CMs in the CPA, it is difficult to make a correct diagnosis for such lesions preoperatively, and therefore they are more likely to be misdiagnosed as other cystic tumors, such as cystic acoustic neuroma, glioma, and hemangioblastoma [15-17]. After reviewing the imaging features of the five cases of cystic CMs in the CPA, we suggest that a diagnosis of cystic CMs may be suspected when a cystic lesion with no involvement of the internal auditory canal and skull base dura is present in the CPA.

Due to the small space of the CPA and the complex surrounding anatomical structures, the presence of CMs will affect the root of the 5^th^-11^th ^cranial nerves, the cerebellum, and the brain stem and result in clinical symptoms. The five cases in the present study presented with symptoms involving the trigeminal nerve, facial and acoustic nerves, and the cerebellum. However, they did not show symptoms of brain stem compression, which may be because the CM likely grows toward the CPA cistern. The trigeminal nerve, facial and acoustic nerves, and the cochlear nerve are quite sensitive, thus even a small CM may cause pronounced clinical symptoms. Therefore, surgical resection is indicated for such CMs. The five patients with cystic CMs described herein underwent surgical resection via a suboccipital retrosigmoid approach with cranial nerve monitoring. Particular care was taken to protect facial and acoustic nerves and the brain stem from injury so as to avert serious postoperative complications. We found that the CMs arising in the CPA adhered to cranial nerves, the cerebellum, the brain stem, and arteries. However, the adhesion seemed to be well circumscribed to allow separation.

The findings described above are in contrast with solid CMs in the CPA, the majority of which arise in the internal auditory canal and have close adhesion with the 7^th ^and 8^th ^cranial nerves. It is quite difficult to free solid CMs from the closely adhered nerves, and more often than not such operations cause clinical symptoms [4]. In addition to taking into account the surrounding nerves while performing surgical resection for cystic CMs in the CPA, neurosurgeons also need to evaluate the degree of blood supply, as this is another critical factor that determines the success of surgical resection. Of the five cases reported herein, four had a rich blood supply and one had a poor blood supply. In one case of a cystic CM with a rich blood supply, total surgical resection had to be performed in two stages due to copious hemorrhaging during the first attempt.

CMs are benign lesions and show a favorable prognosis after complete resection. However, possible injury to cranial nerves during surgery is directly associated with the surgical outcomes due to the complex structures of the CPA. Two patients experienced an uneventful recovery. In contrast, one patient died and two patients did not show improvement in their symptoms, although they did not develop postoperative complications. The possible causes of the poor outcomes include unavailability of cranial nerve monitoring and limited microsurgical skills in two cases and the failure to completely resect the CM in a single attempt in one case with a rich blood supply.

## Conclusions

In conclusion, although cystic CMs in the CPA have no specific imaging features, neurosurgeons should consider the likelihood of CMs when a cystic lesion with no adhesion to the internal auditory canal and skull base dura mater is present in the CPA. Although cystic CMs also involve cranial nerves, the cerebellum, the brain stem, and arteries, they can be separated from these surrounding structures because of the presence of well-margined adhesions; this trait is not present in solid CMs. Skillful microsurgical techniques and cranial nerve monitoring are two critical factors that can ensure a favorable curative outcome in most cases of cystic CMs in the CPA.

## Consent

Written informed consents were obtained from the patient for publication of this case report and accompanying images. Copies of the written consent are available for review upon request.

## Competing interests

The authors declare that they have no competing interests.

## Authors' contributions

KX wrote the initial draft. HH and KX contributed equally to this work. JY is the surgeon. All authors read and approved the final manuscript.

## References

[B1] Bertalanffy H Benes L Miyazawa T Alberti O Siegel AM Sure U Cerebral cavernomas in the adult. Review of the literature and analysis of 72 surgically treated patients Neurosurg Rev 2002 25 1 53 10.1007/s101430100179 11954761

[B2] Batra S Lin D Recinos PF Zhang J Rigamonti D Medscape: Cavernous malformations: natural history, diagnosis and treatment Nat Rev Neurol 2009 5 659 70 10.1038/nrneurol.2009.177 19953116

[B3] Martin NA Vinters H Barrow DL Pathology and grading of intracranial vascular malformation 1990 Intracranial Vascular Malformation. Park Ridge, IL: AANS 1 30

[B4] Engh JA Kostov D St Martin MB Yeaney G Rothfus W Hirsch B Kassam AB Cavernous malformation tumors: a case study and review of the literature Otol Neurotol 2010 31 294 8 10.1097/MAO.0b013e3181c34bf2 19887972

[B5] Iplikçioğlu AC Benli K Bertan V Ruacan S Cystic cavernous hemangioma of the cerebellopontine angle: case report Neurosurgery 1986 19 641 2 3491342 10.1227/00006123-198610000-00025

[B6] Brunori A Chiappetta F Cystic extra-axial cavernoma of the cerebellopontine angle Surg Neurol 1996 46 475 6.4 10.1016/S0090-3019(96)00153-X 8874549

[B7] Vajramani GV Devi BI Hegde T Srikanth SG Shankar SK Cystic cavernous malformation of the cerebellopontine angle Clin Neurol Neurosurg 1998 100 133 7 10.1016/S0303-8467(98)00016-X 9746302

[B8] Stevenson CB Johnson MD Thompson RC Cystic cavernous malformation of the cerebellopontine angle Case illustration. J Neurosurg 2005 103 931 10.3171/jns.2005.103.5.0931 16305001

[B9] Ohba S Shimizu K Shibao S Nakagawa T Murakami H Cystic cavernous angiomas Neurosurg Rev 2010 33 395 400 10.1007/s10143-010-0245-x 20174956

[B10] Batra S Lin D Recinos PF Zhang J Rigamonti D Medscape: Cavernous malformations: natural history, diagnosis and treatment Nat Rev Neurol 2009 5 659 70 10.1038/nrneurol.2009.177 19953116

[B11] Sato K Kubota T Large calcified cystic cavernous angioma in the thalamus--case report Neurol Med Chir (Tokyo) 1995 35 100 3 10.2176/nmc.35.100 7753307

[B12] Hatashita S Miyajima M Koga N Cystic cavernous angioma--case report Neurol Med Chir (Tokyo) 1991 31 414 6 10.2176/nmc.31.414 1720221

[B13] Pinker K Stavrou I Knosp E Trattnig S Are cerebral cavernomas truly nonenhancing lesions and thereby distinguishable from arteriovenous malformations? MRI findings and histopathological correlation. Magn Reson Imaging 2006 24 631 7 10.1016/j.mri.2005.10.037 16735186

[B14] Hauck EF Barnett SL White JA Samson D Symptomatic brainstem cavernomas Neurosurgery 2009 64 61 70 10.1227/01.NEU.0000335158.11692.53 19050659

[B15] Yagi K Kageji T Nagahiro S Murayama Y Multiple cystic cavernous angiomas associated with hemorrhage Acta Neurochir (Wien) 2005 147 201 3 10.1007/s00701-004-0381-6 15365796

[B16] Tomlinson FH Houser OW Scheithauer BW Sundt TM Jr Okazaki H Parisi JE Angiographically occult vascular malformations: a correlative study of features on magnetic resonance imaging and histological examination Neurosurgery 1994 34 792 9 10.1227/00006123-199405000-00002 8052376

[B17] Pozzati E Acciarri N Tognetti F Marliani F Giangaspero F Growth, subsequent bleeding, and de novo appearance of cerebral cavernous angiomas Neurosurgery 1996 38 662 9 10.1097/00006123-199604000-00006 8692382

